# Chronic Rheumatic Arthritis with Osteophatic Outgrowths

**Published:** 1883-04

**Authors:** J. S. Windolph

**Affiliations:** Student at Columbia Veterinary College


### XI.—CHRONIC RHEUMATIC ARTHRITIS WITH OSTEOPHATIC OUTGROWTHS OCCURRING IN A YOUNG OX.

BY J. S. WINDOLPH,

*Student at Columbia Veterinary College.*

The animal from which this specimen was taken was an ox of the grade known as Shorthorn, color nearly white, with only a few rone spots. The animal, purchased out of a drove of western cattle, was finely proportioned, with the exception of weak and crooked legs.

He was turned into a meadow with others of the same drove, and seemed to do as well as the rest for several months.

The first symptoms were swelling of the joints which were soft to the touch, accompanied by a slight rise in temperature. Later on, the ox became more stiff and seemed to have pain, and a crackling sound was heard when the joint was moved. Still later the hock became somewhat semi-flexed.

The appetite remained fair, yet the animal constantly lost flesh and strength until finally his movements were executed with great difficulty.

A shed was built for him near the barn where he was placed for the winter, the owner thinking he must have broken his leg.

His appetite remained fairly good during the winter, but he did not gain much either in flesh or strength.

In the spring it was with great difficulty that he was removed from his shed to the pasture. For a time he apparently picked up a little, but all the joints began to show marked signs of enlargement, and the one first involved was absolutely immovable.

By fly time the ox was very weak, thin, and perfectly hidebound.

He died towards fall, the most emaciated animal I ever saw.

This particular joint I saved, as it was the one most involved. Most of the other joints of the extremities were more or less disorganized by bony deposits around them, this condition being most marked at the carpal articulations.

The specimen, which is now in the Columbia Veterinary College Museum, is an exceedingly fine example of this form of joint disease. Although the articular surfaces are still free, a complete anchylosis has resulted from the interlocking and ossification of the nodular growth springing from the opposing bones.

[This, in connection with a similar specimen removed from a corresponding joint in the horse, render the museum of the college rich in this form of lesion, as it is in almost every department. Although the museum collection is not as rich in numbers as some older ones, it comprises a very rare collection, and illustrates almost every diseased condition, and also many rare anatomical specimens. The anatomical specimens added by the class of 1883 are unusually fine.—ED.]

